# Minimally Invasive Lumbopelvic Fixation for Unstable U-Type Sacral Fractures

**DOI:** 10.7759/cureus.5621

**Published:** 2019-09-11

**Authors:** Darshan S Shah, Taylor Bates, Justin Fowler, Patrick Osborn, Anton Y Jorgensen

**Affiliations:** 1 Orthopaedic Surgery, San Antonio Military Medical Center, San Antonio, USA

**Keywords:** sacral fracture, minimally invasive lumbar fusion, sacro-iliac, ilio-sacral, u-type sacral fracture, lumbopelvic dissociation

## Abstract

Multi-planar transverse, U-type, and vertical sacral fractures occur from high energy trauma or as pathologic fractures and often have associated neurologic and extremity injuries. Modern treatment algorithms fall into two broad categories: 1) percutaneous posterior pelvic fixation (iliosacral or transiliac-transsacral screws) or 2) lumbopelvic fixation. Posterior pelvic screw fixation is minimally invasive but typically requires restricted weight bearing until fracture union. In many cases, lumbopelvic fixation allows for a closed reduction and provides stability to allow full weight bearing immediately after surgery; however, this fixation is often removed in a second surgery after fracture healing. Lumbopelvic fixation was originally described as an open procedure, minimally invasive lumbopelvic fixation is a recent variation and has shown promising results with less morbidity. We present a case series of unstable U-type sacral fractures treated with minimally invasive lumbopelvic fixation with staged hardware removal to illustrate the advantages and complications associated with this new technique. Ten patients with U-type sacral fractures underwent minimally invasive lumbopelvic fixation from 2016 to 2019. Six patients underwent scheduled hardware removal an average of 3.5 (range 1.9-5.5) months after index surgery. Two patients did not undergo hardware removal due to short life expectancy and diagnosis of pathologic fractures. One patient was lost to follow-up. One patient had failed fracture reduction and went on to sacral malunion that required a late sacral extension osteotomy to restore her ability to stand upright. Final disposition of all nine patients with follow-up was normal standing upright posture and normal ambulation without assistive device. There were no late displacements on postoperative upright radiographs. Complex sacral fractures are a challenging injury that can be treated with percutaneous posterior pelvic or lumbopelvic fixation. Lumbopelvic fixation offers the advantages of closed reduction to restore pelvic incidence and immediate weight bearing but has greater surgical morbidity than percutaneous posterior pelvic fixation and often requires hardware removal. The morbidity of lumbopelvic fixation may be reduced with minimally invasive techniques. Minimally invasive lumbopelvic fixation is a treatment option to be considered for complex sacral fractures.

## Introduction

U-shaped sacral fractures are uncommon injuries that cause dissociation between the lower sacrum/pelvis from the upper sacrum/lumbar spine. These fracture patterns can be difficult to interpret on plain radiographs and early reports without the benefit of advanced imaging reflect the difficulty in understanding the fracture lines within the sacrum [1]. Denis et al. classified the injury in a three-zone system. Zone one sacral fractures have vertical fracture lines traversing the ala. Zone two fractures have vertical fracture lines traversing the foramina. Zone three fractures traverse the central spinal canal and have horizontal and vertical fracture lines.

U-type sacral fractures are zone three injuries that consist of many fracture lines but fundamentally only two main fracture fragments [2]. The superior fragment consists of the upper sacrum attached to the axial skeleton while the inferior fragment consists of the lower sacrum, the sacral ala, and the pelvis. The result is a dissociation between the axial skeleton/upper sacrum and the pelvis/sacral ala/lower sacrum [1,3]. The mechanism of injury is a high energy upward force applied through the hips. The fracture angulation varies according to the direction of force, most commonly the upper sacrum is tipped forward into flexion [3].

The two types of operative fixation for complex sacral fractures are posterior pelvic fixation (iliosacral or transiliac-transsacral screws) and lumbopelvic/triangular fixation. Fluoroscopy and navigation allow the placement of posterior pelvic fixation percutaneously [4]. Lumbopelvic fixation constructs consist of bilateral pedicle screws from L4 or L5 connected by rods to pelvic fixation [5]. The advantages of lumbopelvic fixation over posterior pelvic fixation are immediate weight bearing and the potential to perform reduction maneuvers using the hardware to restore pelvic incidence [6-10]. However, lumbopelvic fixation often requires staged hardware removal. Pelvic incidence is the angular relationship between the base of the axial skeleton and the pelvis, it is a critical parameter in the ability to stand upright [11-13]. Mean pelvic incidence in the normal asymptomatic population is 53˚ with standard deviation of +/-9˚[11]. Unstable U-type sacral fractures with the upper sacrum displaced into flexion have an increased pelvic incidence and are associated with loss of normal ability to stand upright. Surgical treatment must include maintenance or restoration of the pelvic incidence [14].

In this series, we review the outcomes and complications of 10 patients who underwent minimally invasive lumbopelvic fixation to treat unstable U-type sacral fractures. We highlight the advantages and disadvantages of lumbopelvic fixation for the treatment of these complex injuries.

## Case presentation

Methods

**Figure 1:**
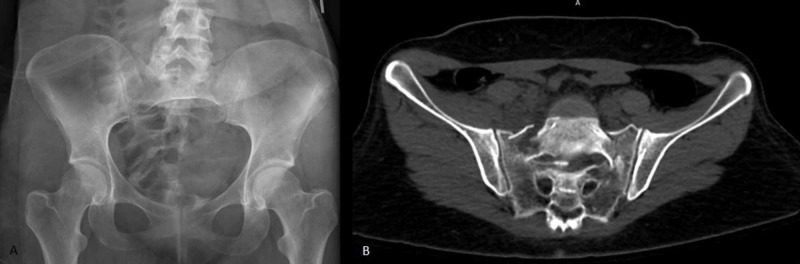
U-type sacral fracture injury films. (A) An AP pelvic radiograph of a U-type sacral fracture. (B) An axial view through the sacrum of a U-type sacral fracture

**Figure 2:**
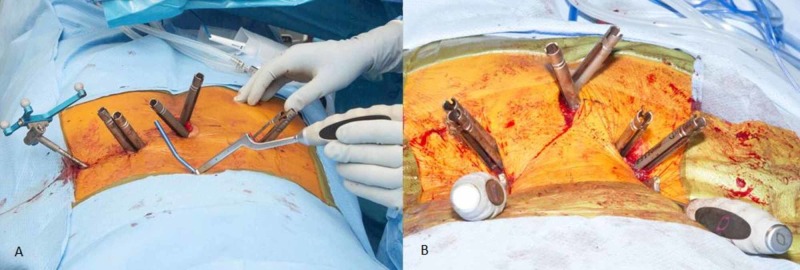
(A )Rod bent using computer control (NuVasive Bendini) attached to rod gripper. (B) Surgical setup for percutaneous instrumentation and reduction. Rods in place with rod grippers sticking out of wound

**Figure 3:**
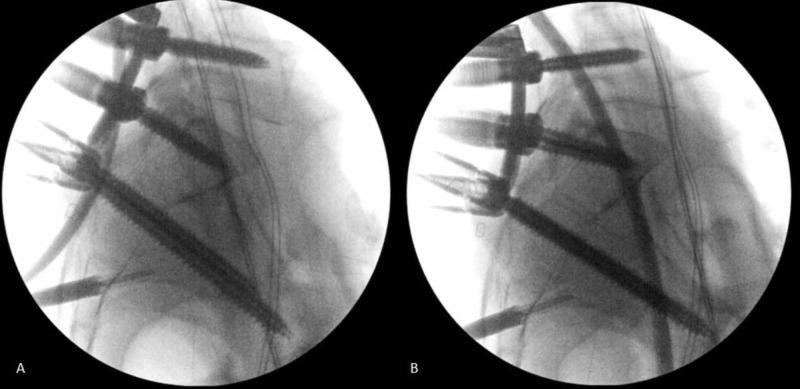
(A and B) Fluoroscopy images showing improvement in sacral kyphosis with reduction maneuver. Lumbopelvic instrumentation can maintain or reduce sacral kyphosis

Ten patients with unstable U-type or vertical sacral fractures were treated with minimally invasive lumbopelvic fixation and fracture reduction (Figure 1A-B). All patients underwent instrumentation with L5 and S1 pedicle screws in the superior fracture fragment and S2 alar iliac screws placed by computer navigation in the inferior fracture fragment. Bilateral 3 cm paramedian incisions were used to place L5 and S1 pedicle screw instrumentation. S2 alar iliac pelvic instrumentation was placed through a single 3 cm midline incision centered over the sacrum (Figure 2A-B). Each S2 alar iliac screw traversed the sacroiliac joint and was placed between the tables of each iliac crest just cephalad to the sciatic notch. Rods were appropriately bent to connect the percutaneous pedicle screws and pelvic bolts using a computer-controlled bender (NuVasive Bendini, San Diego, CA). If necessary, to reduce the fracture, the rod was manually bent into additional lordosis. The rods were then passed under the fascia connecting the incisions and locked into the L5-S1 pedicle screw instrumentation. Grippers attached to the cephalad ends of the rods were used to hyperextend the upper sacral fracture fragment to maintain or restore pelvic incidence. Rod manipulation is performed under fluoroscopic guidance (Figure 3A-B) and the contour of the ventral cortex of the sacrum is used to judge fracture reduction. Rod grippers are firmly grasped and pulled dorsally to tip the L5-S1 levels into hyperextension bringing the caudal end of the rod down into the S2 alar iliac bolts and then locked with set screws. The hyperextension maneuver with the rods locked at L5-S1 is used proportional to the amount of fracture reduction that is required.

**Figure 4:**
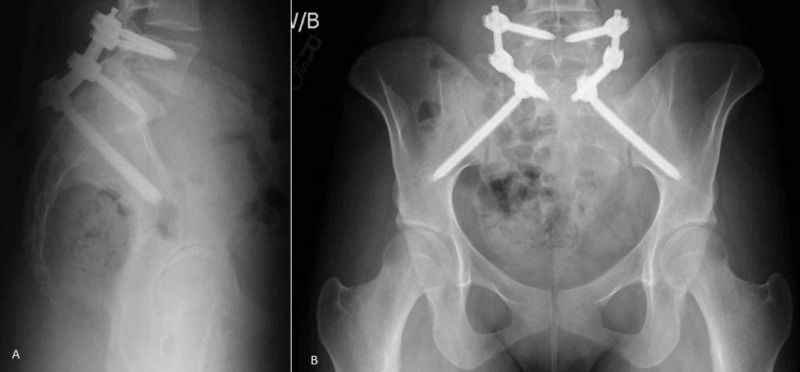
(A) Lateral radiograph of final construct after minimally invasive lumbo-pelvic fixation. Percutaneous fixation was done from L5 to S2. (B) AP radiograph of the minimally invasive lumbo-pelvic fixation. All images are weight bearing

A lateral pelvis fluoroscopy image was checked to ensure that pelvic incidence had been restored. Fractures with excessive impaction or severe angulation can be difficult to reduce with this maneuver, however, every effort should be made to achieve a closed reduction since a sacral malunion requires a complex osteotomy for correction. Non-displaced fractures were instrumented in situ. Patients were mobilized immediately with no weight bearing restrictions. Radiographs were taken immediately post-op and at the 6-week post-op visit to assess maintained alignment (Figure 4A-B). Hardware removal was scheduled 2-3 months after surgery and was performed with fluoroscopic assistance through the same index surgical incisions. Patients were examined in the clinic for wound complications and the ability to stand upright and ambulate. Upright lateral pelvis radiographs were checked after hardware removal to ensure fracture healing and document final pelvic incidence.

Results

Ten patients underwent minimally invasive lumbopelvic fixation from 2016 to 2019. Eight patients had sacral fractures from high energy trauma, and two had pathologic sacral fractures from falls from standing height and did not undergo implant removal due to short life expectancy. One patient with polytrauma including complex neurologic injuries was lost to follow-up and did not have implant removal. Of the eight patients who had planned hardware removal, six underwent scheduled removal an average of 3.5 (range 1.9-5.5) months after index surgery. The hardware removals have been uncomplicated and performed as outpatient surgeries without extending the surgical incisions. One of the planned hardware removal patients was lost in follow-up. The one other planned hardware removal patient had a sacral malunion and required hardware revision and sacral osteotomy. All nine patients who have complete follow-up transitioned to full weight bearing immediately after surgery; final disposition of all patients was normal ambulation without assistive devices, restoration of upright posture, and a final fracture union with no late displacement. All patients with normal ability to ambulate had a restoration of their pelvic incidence to within two standard deviations of normal (Table 1). There were no approach-related complications, wound infections, dural tears, or pseudomeningoceles.

**Table 1:** Lumbopelvic fixation data for 10 patients who underwent MIS fixation for unstable U-type sacral fractures between 2016-2019 at a single institution. Highlighted patients underwent attempted closed reduction.

Patient ID	Age	Closed Reduction (Y/N)	Time to Removal (months)	Complications	Diagnosis	Pre op PI	Post op PI	Final VAS
1	78	N	No removal	None	Pathologic sacral fracture, prostate CA	66˚	69˚	0
2	42	N	4.13	None	Trauma unstable sacral fracture	54˚	47˚	5
3	47	N	1.87	None	Trauma unstable sacral fracture	72˚	69˚	0
4	23	Y	4.03	None	Trauma unstable sacral fracture	57˚	48˚	0
5	23	Y	2.90	None	Trauma unstable sacral fracture	68˚	58˚	0
6	46	N	5.53	None	Trauma unstable sacral fracture	63˚	63˚	4
7	63	N	No removal	None	Polytrauma with sciatic nerve transection	Not measured	54˚	Unknown
8	28	N	2.80	None	Trauma unstable sacral fracture	59˚	68˚	5
9	88	N	No removal	None	Pathologic sacral fracture, end stage renal disease	50˚	59˚	0
10	47	Y	3.47	Cauda Equina/Sacral Malunion	Sacral fracture malunion requiring late osteotomy	109˚	115˚ initial/88˚ after late corrective osteotomy	6

One patient had serious complications of cauda equina syndrome and a sacral malreduction. She required a sacral laminectomy and had a malunion that required a late sacral extension osteotomy to restore her ability to stand upright. This patient was neurologically intact with normal rectal tone upon arrival after the initial injury. She underwent minimally invasive attempted reduction and lumbopelvic fixation. A closed reduction was not achieved, possibly due to fracture impaction or surgeon inexperience as she was early in the case series, and her pelvic incidence was not restored. She had increasing pain, paresthesias, and lower extremity weakness that worsened daily. Serial examinations were performed and the spine surgeon on call noted the loss of rectal tone and severe paresthesias in a saddle distribution five days after injury at which point she underwent a sacral laminectomy. Following the laminectomy her paresthesias resolved and she regained normal bowel and bladder function. Her follow-up X-rays showed a pelvic incidence of 115˚, more than twice the normal of 53˚. On clinical examination, the patient was not able to maintain an upright posture and had great difficulty walking even short distances due to positive sagittal imbalance. She underwent a late sacral extension osteotomy to restore her pelvic incidence and at her last follow-up, she was able to ambulate and stand upright in a normal manner.

## Discussion

Complex unstable sacral fractures are a challenging injury treated with percutaneous posterior pelvic fixation or lumbopelvic fixation. Percutaneous posterior pelvic fixation (transiliac-transsacral or iliosacral screws) allows in situ fixation of the fracture without a reduction maneuver, or may be performed in the prone position to exaggerate lordosis and achieve some fracture reduction. The disadvantages of percutaneous posterior pelvic fixation are the inability to directly manipulate the fracture fragments and insufficient stability for immediate weight bearing. Nork published his experience with a posterior pelvic fixation on 13 patients with unstable U-type sacral fractures where he used bracing and restricted each patient to a wheelchair after surgery for 8-12 weeks [15]. Percutaneous posterior pelvic fixation may not provide sufficient stability in sacral fractures that are vertically unstable. Even with weight bearing restrictions, Griffin and colleagues reported a 13% failure rate of iliosacral fixation in sacral fractures with vertical instability [16].

Open lumbopelvic fixation is associated with high rates of complications including infections, wound complications, dural tears, and pseudomeningocele that may be related to the soft tissue dissection needed to obtain adequate exposure [17]. Our series of minimally invasive lumbopelvic fixation had only one patient with complications which were due to inadequate sacral reduction and malunion. There were no approach related or wound complications which is significantly lower than the 26% rate reported with open lumbopelvic fixation [17,18].

To reduce the sacral fracture and restore normal standing posture the surgeon must understand the relationship between the sacrum and the overall alignment of the spine. Dubousset described the state of balanced upright posture where the knees are fully extended, the hips are in a neutral position, and the pelvis with the rest of the spine are maintained upright within a narrow cone without active muscle contraction [19]. If a malreduced sacral fracture rotates forward into flexion, the lumbar spine must extend into increased lordosis, the hips extend, and the knees flex to maintain the center of gravity over the pelvis [13-14]. These compensations require active muscle contraction and consume energy and are associated with significant disability [13,20]. The pelvic incidence defines the relationship of the sacrum and pelvis to the upright spine [12]. Unstable U-type sacral fractures with the upper sacrum tipped forward into flexion have an increased pelvic incidence. Therefore, restoration of normal pelvic incidence is critical to the ability to maintain a comfortable upright posture [14]. Surgical treatment must include maintenance or restoration of the pelvic incidence [13-14].

Spine surgeons now have a broader understanding of the concepts of sagittal balance and the “cone of economy” [19]. Percutaneous lumbopelvic fixation may help to restore a normal sagittal alignment by allowing manipulation of the fracture through rods attached to pedicle screws at L5 and S1. Even with the use of closed reduction maneuvers with lumbopelvic fixation, an inadequate reduction may still occur if fracture fragments are highly impacted. Great care must be taken to achieve a closed reduction and restore pelvic incidence to prevent complex late revision surgery. In this series one patient had a highly angulated and impacted sacral fracture and closed reduction with lumbopelvic fixation was not successful. Mean pelvic incidence in the normal asymptomatic population is 53˚ with standard deviation of +/-9˚ [11]. In this series, all patients who had a final pelvic incidence within two standard deviations of the mean were able to ambulate and stand with a normal and comfortable upright posture. The one patient who failed closed reduction had a final pelvic incidence of 115˚ and could not stand upright, she required a complex late sacral extension osteotomy to restore a normal sagittal alignment.

This series of patients illustrates the benefits and the complications of minimally invasive lumbopelvic fixation for complex sacral fractures. The advantages of lumbopelvic fixation include immediate full weight bearing. Lumbopelvic fixation can be used to maintain or to restore pelvic incidence. Our series is similar to previous reports that show minimally invasive lumbopelvic fixation for unstable sacral fracture is an option in treating these complex injuries. The minimally invasive technique is associated with lower wound complication rates compared to open lumbopelvic fixation and still allows for immediate full weight bearing [5-8]. Every effort should be made to reduce the sacral fracture at the index surgery to prevent a sacral malunion.

## Conclusions

When closed reduction and normal pelvic incidence was achieved, patients in this series had good clinical outcomes. When the pelvic incidence was not restored, as in our one patient, the clinical outcome was poor. If the surgeon carefully considers the restoration of pelvic incidence then minimally invasive closed reduction and lumbopelvic fixation is a viable treatment option for complex unstable sacral fractures.
